# Gene Therapy for Human Lung Adenocarcinoma Using a Suicide Gene Driven by a Lung-Specific Promoter Delivered by JC Virus-Like Particles

**DOI:** 10.1371/journal.pone.0157865

**Published:** 2016-06-20

**Authors:** Chun-Nun Chao, Mien-Chun Lin, Chiung-Yao Fang, Pei-Lain Chen, Deching Chang, Cheng-Huang Shen, Meilin Wang

**Affiliations:** 1 Institute of Molecular Biology, National Chung Cheng University, Chiayi, Taiwan; 2 Department of Pediatrics, Ditmanson Medical Foundation Chiayi Christian Hospital, Chiayi, Taiwan; 3 Department of Urology, Ditmanson Medical Foundation Chiayi Christian Hospital, Chiayi, Taiwan; 4 Department of Medical Research, Ditmanson Medical Foundation Chiayi Christian Hospital, Chiayi, Taiwan; 5 Department of Medical Laboratory Science and Biotechnology, Central Taiwan University of Science and Technology, Taichung, Taiwan; 6 Department of Health and Nutrition Biotechnology, Asia University, Taichung, Taiwan; 7 Department of Microbiology and Immunology, Chung Shan Medical University, Taichung, Taiwan; Swedish Neuroscience Institute, UNITED STATES

## Abstract

Lung adenocarcinoma, the most commonly diagnosed type of lung cancer, has a poor prognosis even with combined surgery, chemotherapy, or molecular targeted therapies. Most patients are diagnosed with an in-operable advanced or metastatic disease, both pointing to the necessity of developing effective therapies for lung adenocarcinoma. Surfactant protein B (SP-B) has been found to be overexpressed in lung adenocarcinoma. In addition, it has also been demonstrated that human lung adenocarcinoma cells are susceptible to the JC polyomavirus (JCPyV) infection. Therefore, we designed that the JCPyV virus-like particle (VLP) packaged with an SP-B promoter–driven thymidine kinase suicide gene (pSPB-tk) for possible gene therapy of human lung adenocarcinoma. Plasmids expressing the GFP (pSPB-gfp) or thymidine kinase gene (pSPB-tk) under the control of the human SP-B promoter were constructed. The promoter’s tissue specificity was tested by transfection of pSPB-gfp into A549, CH27, and H460 human lung carcinoma cells and non-lung cells. The JCPyV VLP’s gene transfer efficiency and the selective cytotoxicity of pSPB-tk combined with ganciclovir (GCV) were tested *in vitro* and in a xenograft mouse model. In the current study, we found that SP-B promoter–driven GFP was specifically expressed in human lung adenocarcinoma (A549) and large cell carcinoma (H460) cells. JCPyV VLPs were able to deliver a GFP reporter gene into A549 cells for expression. Selective cytotoxicity was observed in A549 but not non-lung cells that were transfected with pSPB-tk or infected with pSPB-tk–carrying JCPyV VLPs. In mice injected with pSPB-tk–carrying JCPyV VLPs through the tail vein and treated with ganciclovir (GCV), a potent 80% inhibition of growth of human lung adenocarcinoma nodules resulted. The JCPyV VLPs combined with the use of SP-B promoter demonstrates effectiveness as a potential gene therapy against human lung adenocarcinoma.

## Introduction

Lung cancer is the leading cause of mortality due to malignancy worldwide [1]. Non–small cell lung cancer (NSCLC) accounts for approximately 80% of lung cancer cases diagnosed. The main types of NSCLC, classified by histology, are squamous cell carcinoma, large cell carcinoma, and adenocarcinoma. Adenocarcinoma is the most common type of lung cancer seen in non-smokers and women, and the relative incidence of adenocarcinoma has risen dramatically in recent decades [2]. The treatment of choice for early-stage NSCLC is surgical resection supplemented by adjuvant cisplatin-based chemotherapy, which improves the patients’ 5-year survival, but by only 4–5% [3]. Although surgery is the best possible treatment, only 20–25% of NSCLC patients are suitable for potentially curative resection [4]. Patients with advanced lung cancer, who are not good candidates for resection, generally have a poor prognosis or eventually develop resistant disease even when treated with newer chemotherapeutic agents or molecular targeted therapies [5–7]. With an overall 5-year survival rate of less than 15% [8], these patients are clearly in need of new, effective therapeutic options.

Virus-like particles (VLPs) are made of viral structural proteins, but are non-virulent because they do not contain viral genomes. The ability of VLPs to package exogenous DNA makes them promising vectors for gene therapy [9]. The JC virus (JCPyV) is a human polyomavirus that causes asymptomatic infection in most adult population and progressive multifocal leukoencephalopathy in AIDS patients [10]. JCPyV enters a human host through the tonsillar stromal tissue of the respiratory tract [11] and persists in kidney and lymphoid tissues during latency [12, 13]. Terminal α (2–6)-linked sialic acid, a critical component of the JCPyV receptor [14], is abundantly expressed in human lung [15]. These findings suggest that the lung might be susceptible to JCPyV infection. Recent reports of the presence of JCPyV DNA and protein in human lung carcinomas [16, 17] indicate that JCPyV may infect human lung carcinoma cells. The major capsid protein of JCPyV, VP1, has been expressed in *E*. *coli* [18], yeast [19], and insect cells [20] and is able to self-assemble into VLPs (JCPyV VLPs) in each of these systems. Furthermore, JCPyV VLPs are capable of packaging exogenous DNA during the assembly process, keeping the DNA well protected, and delivering this DNA with high efficiency into JCPyV susceptible cells for expression [21–23]. Thus, JCPyV VLPs may be used for delivering genes into human lung carcinoma cells for therapeutic purposes.

Gene therapy for cancer is the use of gene delivery vectors that can navigate to tumor cells and allow therapeutic genes to be specifically expressed in tumor cells [24]. As a potential gene therapy for cancer, combining JCPyV VLPs with cancer specific promoters could greatly improve their tissue specificity. Surfactant protein B (SP-B) is a hormonally regulated lung protein that plays important roles in surfactant function and homeostasis [25]. Because the expression of SP-B is restricted to alveolar type II cells and Clara cells of the lung, the SP-B promoter may be used for gene therapy of lung carcinomas [26]. In this study, we tested the tissue selectivity of the SP-B promoter by using it to drive the expression of a reporter gene or a suicide gene in lung carcinoma cells. The ability of JCPyV VLPs carrying a suicide gene driven by the human SP-B promoter to target lung adenocarcinoma tumors in an animal model was also assessed.

## Materials and Methods

### Cell lines

A549 human lung adenocarcinoma cells (ATCC, CCL-185) were maintained in Dulbecco's modified Eagle's medium (DMEM) (Gibco, Cat. No. 1230–032) supplemented with 10% fetal bovine serum (FBS) and antibiotics (100 U/ml penicillin and 100 μg/ml streptomycin). CH27 human lung squamous carcinoma cells (a gift from J. T. Chang [27]) were grown in monolayer culture in DMEM containing 10% FBS, antibiotics, 2 mM glutamine, 1.5 g/L sodium bicarbonate, and 0.1 mM non-essential amino acids. H460 human lung large cell carcinoma cells (ATCC, HTB-177) were maintained in RPMI 1640 medium with 2 mM L-glutamine and adjusted to contain 10 mM HEPES, 1 mM sodium pyruvate, 4.5 g/L glucose, 1.5 g/L sodium bicarbonate, and 10% FBS. IMR32 human neuroblastoma cells (ATCC, CCL-127) were cultured in 90% Eagle’s minimum essential medium with Earle's balanced salt solution, 2 mM L-glutamine, 1.5 g/L sodium bicarbonate, 0.1 mM non-essential amino acids, and 1.0 mM sodium pyruvate and 10% heat-inactivated FBS. HK-2 cells (ATCC, CRL-2190), a line of human kidney proximal tubular cells immortalized by human papillomavirus type 16 E6/E7 genes, were maintained in 1:1 DMEM:Ham F12 (Gibco, Cat. No. 11765–054) supplemented with 5 μg/mL transferrin (Merck, Cat. No. 616424), 5 μg/mL insulin (Sigma-Aldrich, St. Louis, MO, Cat. No. I-1882), 400 ng/mL hydrocortisone (Sigma-Aldrich, Cat. No. H-0315), 5 ng/mL sodium selenite (Sigma-Aldrich, Cat. No. S-9133), 10% FBS, and antibiotics.

### Construction of expression plasmids containing the SP-B promoter

The human SP-B promoter fragment was amplified from the genomic DNA of A549 cells by PCR using the primers 5'-GGGCCCACATGTATAGGGCTGTCTG-3' and 5'-CCGTCGACGCATGGCCCCTTATAGC-3'. The PCR product was digested with PciI and SalI (NEB, Ipswich, MA) and ligated using T4 DNA ligase (NEB) with pEGFP-N1 plasmid DNA (Clontech Laboratories, Inc., Mountain View, CA) from which the CMV promoter had been removed by digestion with PciI and SalI. The resulting plasmid, pSPB-gfp, was confirmed by DNA sequencing. The SP-B promoter PCR product described above was also digested with SpeI and SalI (NEB) and ligated using T4 DNA ligase with pUMVC1-tk plasmid DNA (Aldevron, Fargo, ND) from which the CMV promoter had been removed by digestion with SpeI and SalI. The resulting plasmid, pSPB-tk, was confirmed by DNA sequencing.

### Production of JCPyV VLPs packaged with expression plasmids

The JCPyV VP1 expression plasmid ΔpFlag-JCPyVP1 [18] was co-transformed with one of pEGFP-N1, pUMVC1-tk, and pSPB-tk into JM109 *E*. *coli* (Promega, Madison, WI). Plasmid pFlag was purchased from Promega (Madison, WI). VLPs packaged with pEGFP-N1, pUMVC1-tk, and pSPB-tk are referred to as gfp-VLPs, CMVtk-VLPs, and SPBtk-VLPs. The procedures for preparing and purifying gfp-VLPs and tk-VLPs were described by Chen *et al*. [22]. Briefly, the transformed *E*. *coli* strains were grown in LB medium with ampicillin and kanamycin to select for ΔpFlag- JCPyVP1 and pEGFP-N1, pUMVC1-tk, or pSPB-tk respectively. VP1 protein expression was induced by the addition of 0.5 mM (final concentration) isopropyl β-D-1-thiogalactopyranoside (IPTG) for 8 h at 30°C. For VLP purification, the supernatant of the *E*. *coli* lysate was collected and subjected to 20% sucrose cushion centrifugation and CsCl velocity gradient centrifugation. Fractions with hemagglutination activity were collected and dialyzed against Tris-buffered saline (TBS) (10 mm Tris-HCl, pH 7.4, 150 mm NaCl). The VLPs were concentrated using a Centricon filter (Millipore, Billerica, MA, USA).

### Transfection and Pseudoinfection

Transfection of cells with various expression plasmids was performed with Lipofectamine 2000 reagent according to the manufacturer’s instructions (Invitrogen, Carlsbad, CA). Briefly, cells were cultured in 35 mm dishes in complete medium. Four μg of plasmid DNA was mixed with 10 μl of Lipofectamine 2000 reagent, and the mixture was incubated for 20 minutes at room temperature and then added to a dish of cells. Six hours later, the culture medium was changed to fresh complete medium, and the cells were incubated at 37°C and 5% CO_2_ for 72 hours.

For pseudoinfection, the cells in 35 mm dishes were washed with cold PBS and incubated with 10 μg of VLPs at 4°C for 1 hour. Afterwards, the cells were washed twice with cool PBS to remove free VLPs, placed in complete medium, and then incubated at 37°C and 5% CO_2_ for 72 hours.

### Cytotoxicity assay

Cell Counting Kit-8 (CCK-8) (Dojindo Laboratories, Kumamoto, Japan) was used to assess the cytotoxic activity of the thymidine kinase suicide gene as follows. Cells were seeded in 96-well flat-bottom microtiter plates (BD Biosciences Clontech, San Diego, CA) at 2 × 10^3^ cells per well and either transfected with DNA at 0.2 μg per well or pseudoinfected with tk-VLPs at 0.4 μg per well. The cells were maintained at 37°C and 5% CO_2_ with or without treatment with 10 μg/ml ganciclovir (GCV) (Cymeven; Roche, Palo Alto, CA). At 72 hours posttransfection or postinfection, the culture medium was changed to 100 μl per well of fresh medium containing 10 μl of CCK-8 solution, and the cells were incubated at 37°C and 5% CO_2_ for 1 hour. Absorbance at 450 nm was then measured using a microplate reader (Biotek Instruments, Winooski, VT).

### Assessment of inhibition of tumor growth by SPBtk-VLPs in a mouse model

The Institutional Animal Care and Use Committee of Chung Shan Medical University approved the animal experiments (Permit Number: 1554). All animal procedures were performed according to approved protocols and in compliance with the recommendations for proper care and use of laboratory animals. Four-week-old male nude mice were purchased from BioLASCO Taiwan Co., Ltd. for experiments. To induce tumor nodule formation, 1 × 10^7^ A549 cells were injected subcutaneously into the right flanks of mice. Two weeks later, the mice were randomized into four groups of four mice each and subjected to treatment with either SPBtk-VLPs with or without GCV, or PBS with or without GCV. SPBtk-VLPs or PBS was administered by tail vein injection at 105 μg per injection once every 3 days. SPBtk-VLPs were diluted with 100 ul of PBS. GCV was given by intraperitoneal injection at 300 mg/kg once every 3 days, six times in total, after the first intravenous injection of SPBtk-VLPs or PBS. GCV was diluted in distilled water with a 10 mg/ml concentration. Mice were sacrificed by cervical dislocation at the end of the treatment period.

### Delivery of gfp-VLP to tumor nodule in mouse

A549 cells were injected subcutaneously into the right flank of a mouse as described above. gfp-VLP were injected through tail vein every other day for 6 times in total. The mouse was anesthetized and the tumor nodule was removed and embedded in optimum cutting temperature compound (Sakura Finetek USA, Inc., Torrance, CA). Cryosectioning was performed to give slices of 10 μm thickness. Fluorescent protein expression was detected with a ZEISS AXioskop2 upright fluorescence microscope (Carl Zeiss, Thornwood, NY).

### Statistical analysis

Data were expressed as mean ± standard deviation. Analysis of data was performed using Student’s *t*-test and one-way ANOVA tests, with a *P* value <0.05 being considered to represent a significant difference.

## Results

### Specific expression of a SP-B promoter–driven reporter gene

**Fig 1:**
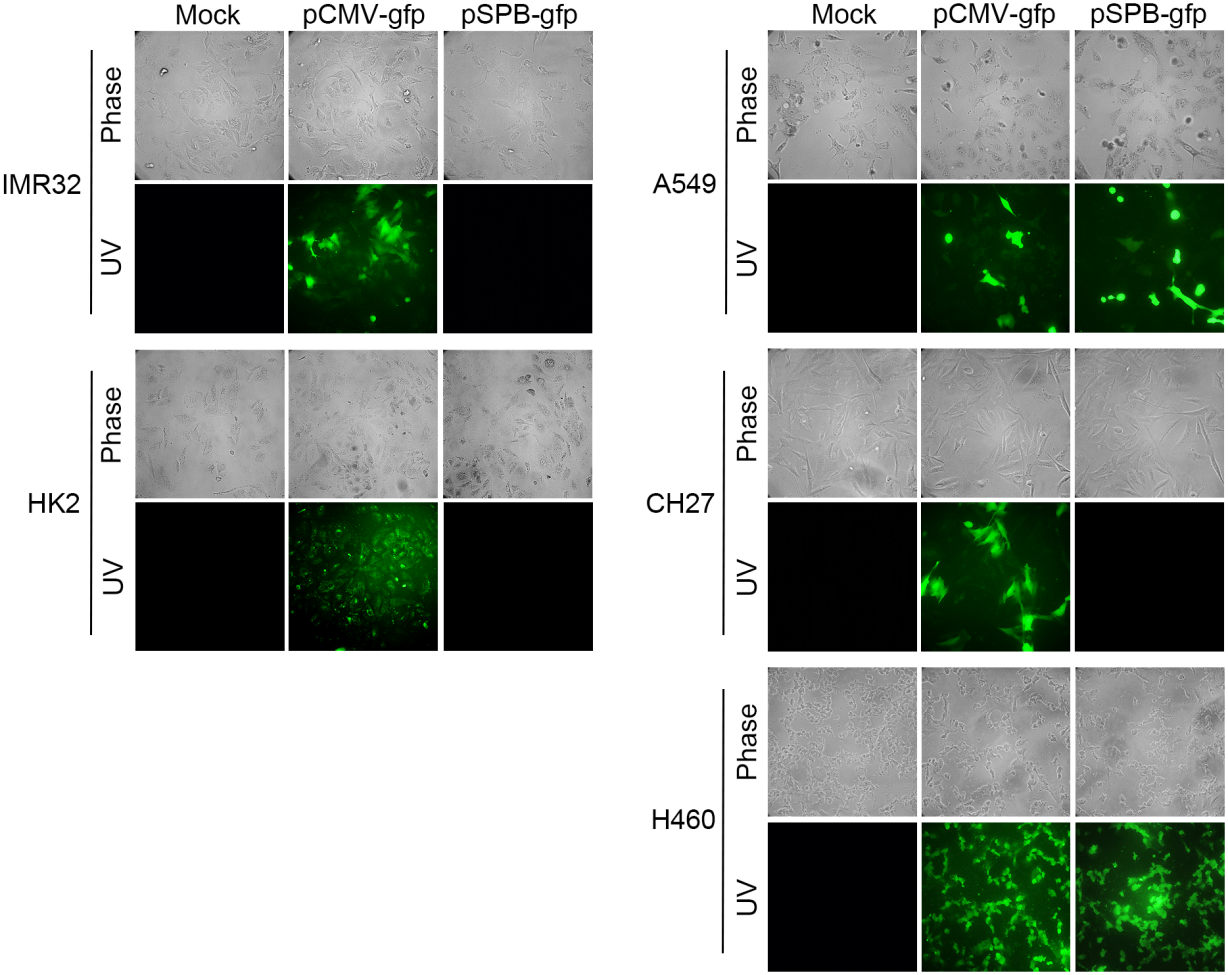
EGFP expression in human lung carcinoma (A549 and H460) cells transfected with pSPB-gfp. IMR32, HK2, and three types of NSCLC cells (A549, CH27, and H460) were transfected with mock, pEGFP-N1 (pCMV-gfp), or pSPB-gfp DNA. The expression of green fluorescent protein in the transfected cells was visualized with a fluorescence microscope. Photos were taken using ZEISS AXioskop2 upright fluorescence microscope (Carl Zeiss, Thornwood, NY) with single-lens reflex camera. Plan-Neofluar class objective was 20X. Magnification was 200X and exposure time was 0.2 seconds. QCapturePro7 software was used for image analysis.

To test the tissue specificity of the SP-B promoter, three types of NSCLC cells, A549, CH27, and H460, and two non-lung cells, IMR32 and HK2, were transfected with pSPB-gfp DNA. GFP expression in the cells was examined 72 hours after transfection. As shown in Fig 1, the SP-B promoter–driven EGFP reporter gene was expressed only in human lung adenocarcinoma (A549) and human lung large cell carcinoma (H460) cells, demonstrating the SP-B promoter’s lung tissue specificity.

### Determination of selective cytotoxic activity of pSPB-tk

**Fig 2:**
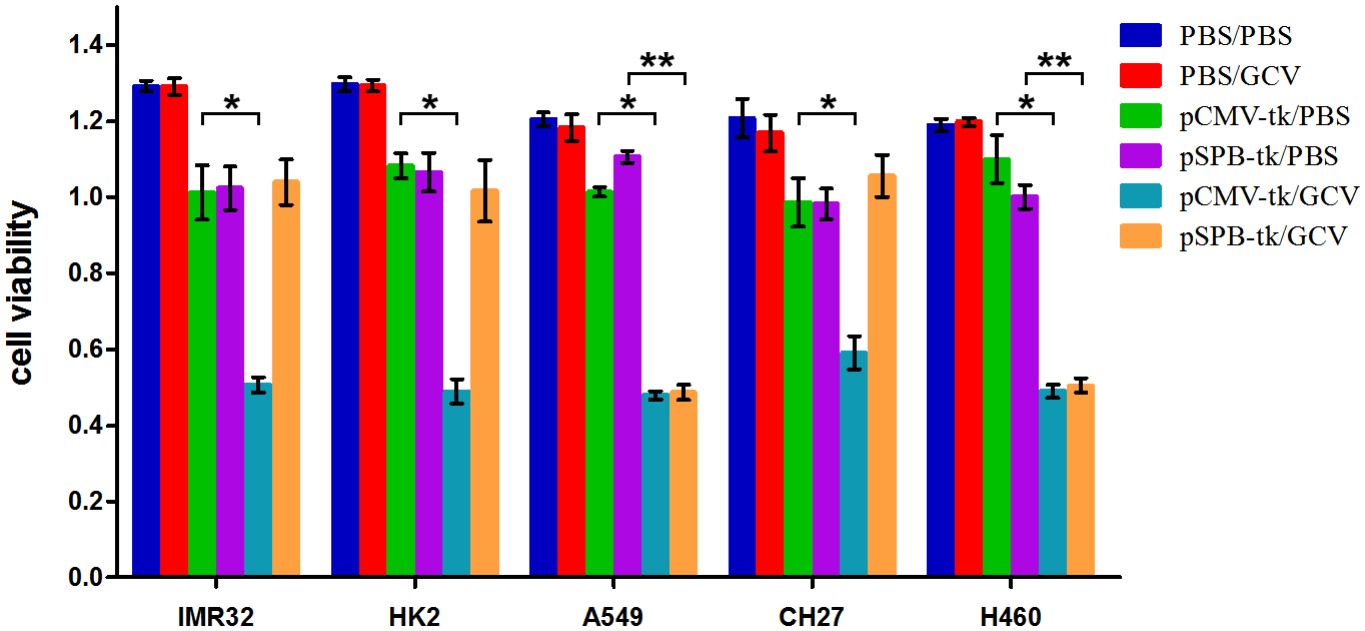
Selective cytotoxic activity of GCV in pSPB-tk–transfected A549 human lung adenocarcinoma cells. The viability of human cells—A549, H460, CH27, IMR32, and HK2—was assessed by the CCK-8 method 72 hours after various treatments. The treatment combinations included PBS followed by PBS (PBS/PBS), PBS followed by GCV (PBS/GCV), pUMVC1-tk followed by PBS (pCMV-tk/PBS), pSPB-tk followed by PBS (pSPB-tk/PBS), pUMVC1-tk followed by GCV (pCMV-tk/GCV), and pSPB-tk followed by GCV (pSPB-tk/GCV). * and ** *P* value <0.005.

To demonstrate that the plasmid pSPB-tk is functional, we carried out transfection followed by a cytotoxicity assay. A promoter–suicide gene construct, either pUMVC1-tk or pSPB-tk, was transfected into NSCLC (A549, H460, CH27) and non-lung (IMR32 and HK2) cells. The transfected cells were treated with GCV and then compared for the extent of cytotoxicity. As seen in Fig 2, pSPB-tk was specifically cytotoxic in A549 and H460 cells but not in CH27, IMR32 and HK2 cells. DNA transfection may usually cause cell cytotoxicity. The cells transfected with pSPB-tk without GCV also showed minor cytotoxic effect probably due to a cytotoxicity from DNA transfection. Taken together, these results showed that the cloned SP-B promoter confers tissue-specific cytotoxicity in A549 and H460 cells.

### Delivery of a reporter gene into A549 cells by JCPyV VLPs

**Fig 3:**
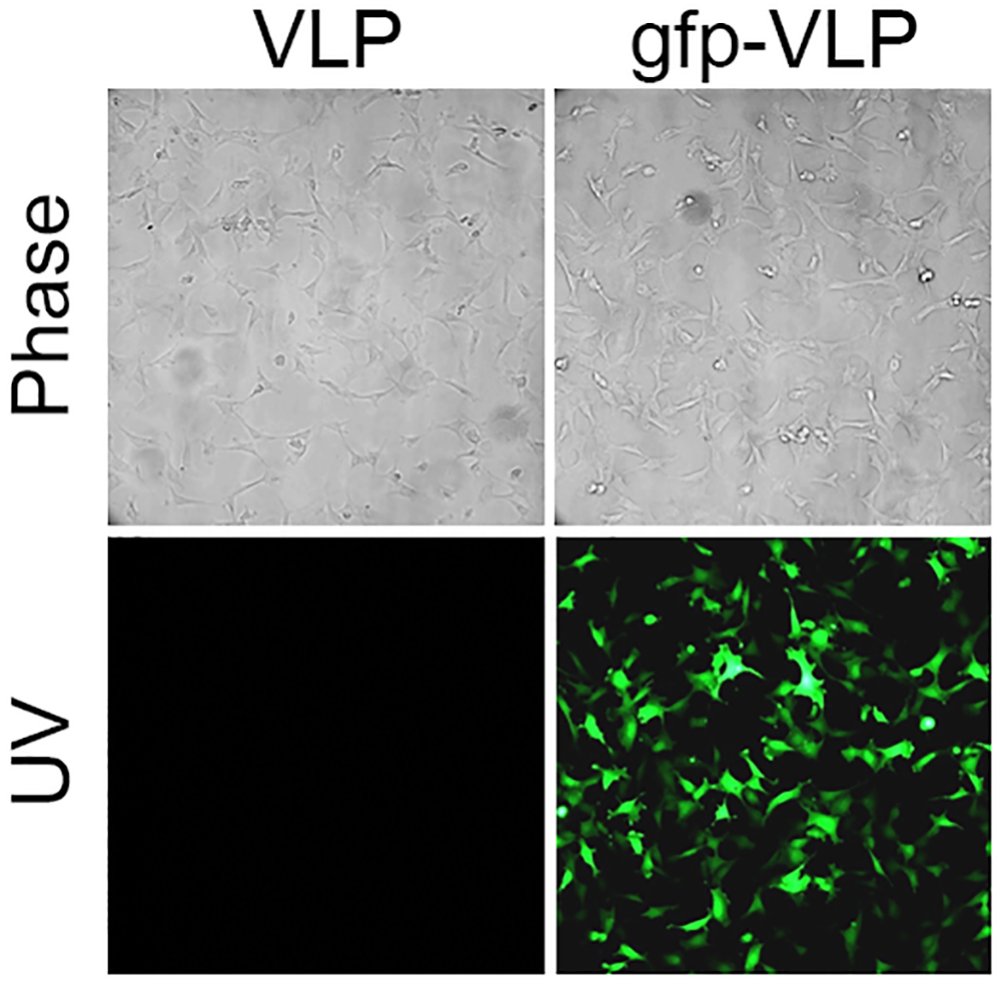
Transduction of the EGFP gene into A549 human lung adenocarcinoma cells by JCPyV VLPs *in vitro*. A549 cells were infected with control VLPs or with gfp-VLPs. The expression of green fluorescent protein in the infected cells was visualized with a fluorescence microscope. Photographing details are the same as described in the legend of Fig 1.

To demonstrate the JCPyV VLP is able to deliver an exogenous DNA into human lung adenocarcinoma, A549 cells were infected with gfp-VLPs. At 72 hours postinfection, gfp expression was detected by fluorescence microscopy. As shown in Fig 3, gfp-VLPs not only entered the target cells but also expressed the gfp gene in almost all A549 cells. This result indicates the feasibility of using the JCPyV VLP as a vector for delivery of exogenous genes to A549 cells for expression.

### Selective cytotoxicity of SPBtk-VLPs

**Fig 4:**
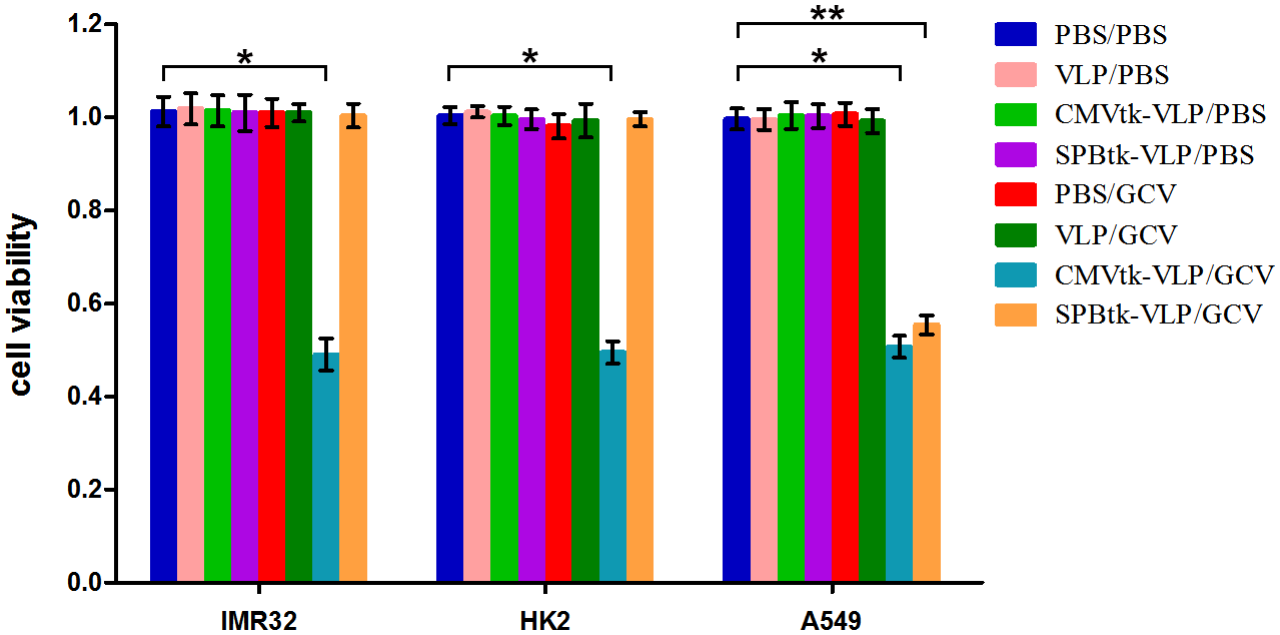
Selective cytotoxicity of GCV to SPBtk-VLP–infected A549 cells. The viability of human cells—IMR32, HK2, and A549—was assessed by the CCK-8 method 72 hours after various treatments. The treatment combinations included PBS followed by PBS (PBS/PBS), control VLPs followed by PBS (VLP/PBS), pUMVC1-tk–packaged VLPs followed by PBS (CMVtk-VLP/PBS), pSPB-tk–packaged VLPs followed by PBS (SPBtk-VLP/PBS), PBS followed by GCV (PBS/GCV), control VLPs followed by GCV (VLP/GCV), pUMVC1-tk–packaged VLPs followed by GCV (CMVtk-VLP/GCV), and pSPB-tk–packaged VLPs followed by GCV (SPBtk-VLP/GCV). * and ** *P* value <0.005.

To demonstrate the specific cytotoxicity of SPBtk-VLPs in A549 cells, IMR32, HK2, and A549 cells were infected with SPBtk-VLPs in the presence of GCV. As shown in Fig 4, SPBtk-VLPs exerted a selective cytotoxic effect only on A549 cells, whereas CMVtk-VLPs were cytotoxic to all three cell types in the presence of GCV. No cytotoxic effect was observed with either type of tk-VLPs alone or with GCV treatment alone. These results show that JCPyV VLPs are able to package and deliver pSPB-tk to lung adenocarcinoma cells and lead to a highly selective cytotoxic effect.

### Inhibition of lung adenocarcinoma growth by SPBtk-VLPs in a xenograft mouse model

**Fig 5:**
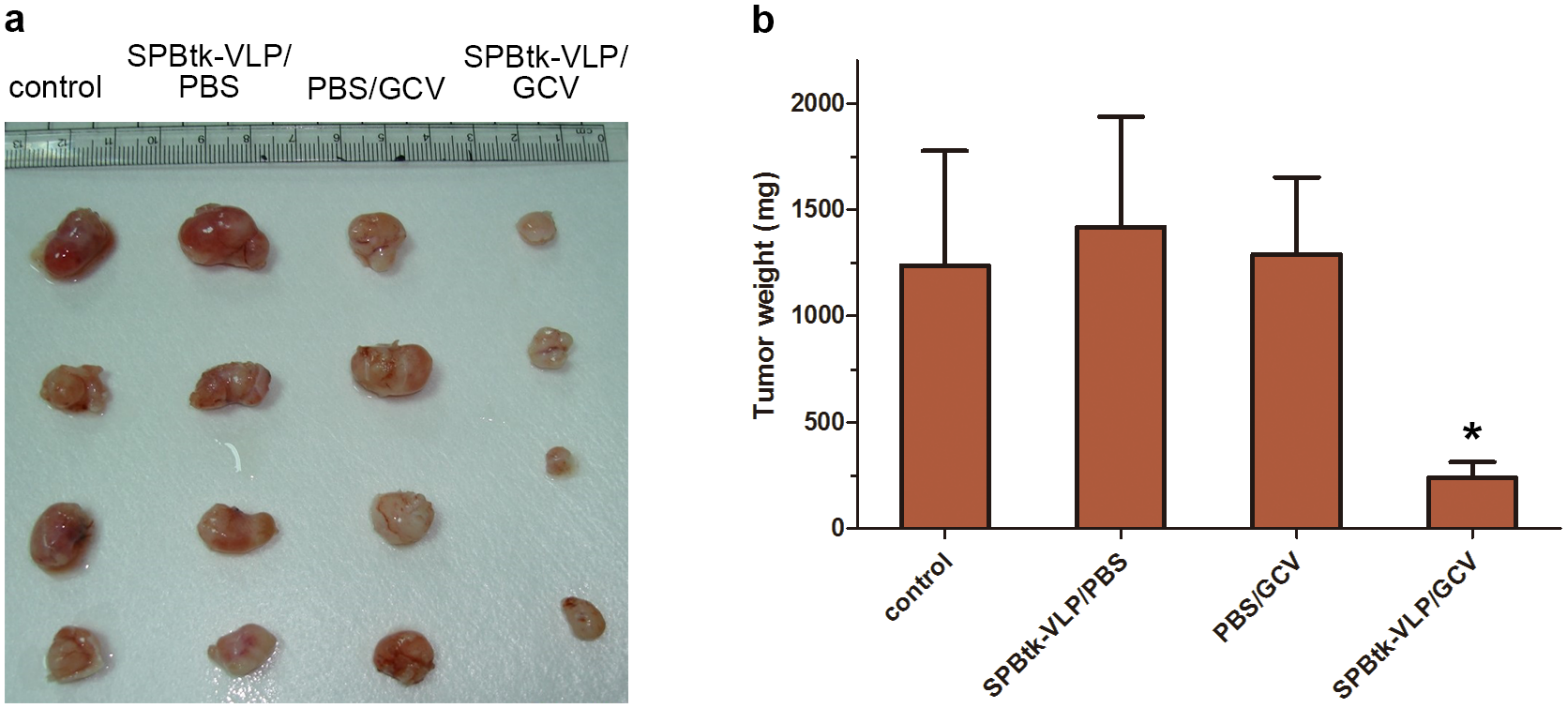
Inhibition of growth of human lung adenocarcinoma tumor nodules by SPBtk-VLPs/GCV in a xenograft mouse model. Human lung adenocarcinoma (A549 cell)–xenografted mice were intravenously administered with PBS or with SPBtk-VLPs, followed by GCV or PBS injection. (**a)** Gross picture of tumor nodules from all treatment groups. (**b)** Quantification of tumor nodule weights. *P < 0.05.

To test that SPBtk-VLP is able to target lung adenocarcinoma through the systemic circulation in a xenograft mouse model, nude mice were subcutaneously injected with A549 cells, which over 2 weeks developed into tumor nodules. The mice were then administered SPBtk-VLPs by tail vein injection and treated with GCV by intraperitoneal injection. As revealed by our results in Fig 5, the mean weight of the tumor nodules at the end of the experiment was 1.25 g or more for each of the control groups lacking either SPBtk-VLPs, GCV, or both, but was 0.25 g for the SPBtk-VLP/GCV group, amounting to at least an 80% inhibition of tumor growth for this treatment group.

### Delivery of gfp-VLP to tumor nodule in mouse

**Fig 6:**
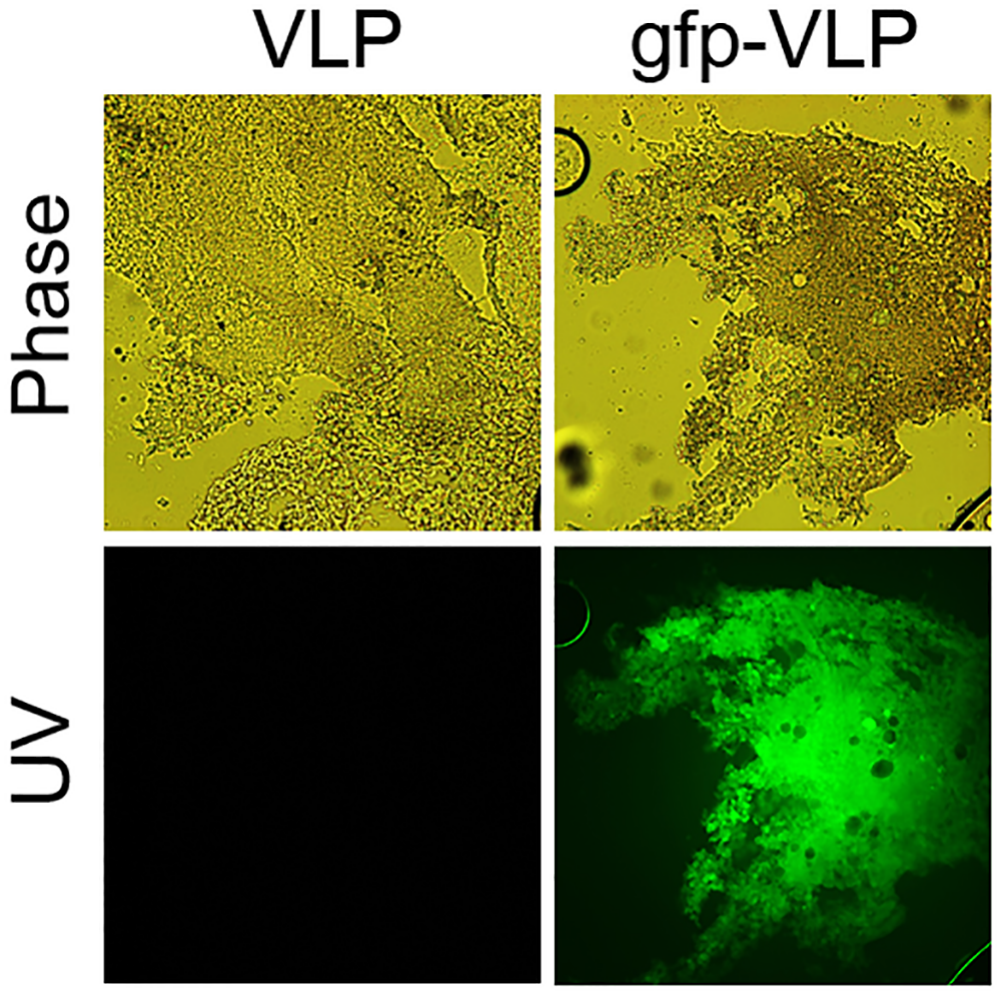
Delivery of gfp-VLP to tumor nodule in mouse. A549 cells were inoculated subcutaneously into mice and followed by injection of either control VLPs or gfp-VLPs through the tail vein. Tumor nodule from each mouse was cryosectioned and examined by fluorescence microscopy.

To demonstrate if the tail-vain injected VLPs could indeed reached the tumor cells in mouse, A549 cells were injected subcutaneously into the right flank of mouse followed by injection gfp-VLPs through tail vein. The tumor nodule was removed and cryosectioned for gene delivery examination. The results revealed that the green fluorescence protein was expressed in the tumor nodule (Fig 6). This finding demonstrated that the VLPs were able to deliver an expression plasmid into the human lung adenocarcinoma cells in the mouse through tail vein injection.

## Discussion

In this study, we demonstrated the tissue specificity of the SP-B promoter in two human lung carcinoma cell lines, A549 adenocarcinoma and H460 large cell carcinoma cells. Although IMR32 and HK2 cells are also JCPyV VLP susceptible cells, the combination of SPBtk-VLPs and GCV had no cytotoxic effect on these two cell types. In our animal model, SPBtk-VLPs administered by tail vein injection were able to travel via the systemic circulation to reach subcutaneous lung adenocarcinoma nodules and to inhibit the growth of the tumors by more than 80%. These results support the potential usefulness of the SP-B promoter–driven suicide gene carried by JCPyV VLPs as a gene therapy strategy for treating human lung adenocarcinoma.

Recently, circulating pro–SP-B is reported to be a biomarker for early detection of NSCLC predominantly in lung adenocarcinoma [28]. It has also been demonstrated that SP-B is overexpressed in primary lung adenocarcinomas as compared with normal lung tissues [29, 30]. Therefore, the SP-B promoter could be used as lung tissue–specific promoter for conferring lung cancer–specific targeting in gene therapy [26, 31]. When the SP-B promoter was used to drive expression of adenovirus E4, viral replication and cell killing by this viral vector were restricted to H441 human lung adenocarcinoma cells *in vitro*; intratumoral injection of this viral vector inhibited the growth of H441-xenografted tumors in nude mice [32]. Therefore, the SP-B promoter was employed for targeting lung cancer gene therapy to take advantage of the promoter’s strict lung tissue specificity in this study. Moreover, we chose the HSV-TK/GCV suicide gene therapy strategy, which has the advantage of being cell cycle–dependent, affecting only dividing cells [33]. Therefore, using the SP-B promoter to drive the thymidine kinase suicide gene not only allows lung adenocarcinoma cells to be targeted transcriptionally but also allows the suicide gene to have a greater impact on cancer cells and be less damaging to normal lung tissue. In a previous study, pro–SP-B was detected in 57% of lung adenocarcinomas and 20% of large cell carcinomas as demonstrated by immunoreactivity [34]. In the current study, we found that transfection with pSPB-gfp led to green fluorescent protein expression in not only A549 adenocarcinoma but also H460 large cell carcinoma cells (Fig 1). It has been reported that SP-B is specifically expressed in type II cells (A549) and large cells (H460) but not in squamous cell carcinoma (CH27) [34, 35]. Therefore, CH27 cells did not express GFP under the control of SP-B promoter. This result indicates that the SP-B promoter is active in a subgroup of large cell lung carcinomas, and suggests that the SP-B promoter can be used to target this subgroup at the transcriptional level for future lung cancer therapy.

JCPyV VLPs can be easily generated in an *E*. *coli* expression system in large quantities and at low cost [18]. Exogenous genes of interest up to 9.4 kbp in size can be packaged by JCPyV VLPs in *E*. *coli* [9] and then delivered into JCPyV susceptible cells with high gene transduction efficiency [36]. In the current study, almost all human lung adenocarcinoma cells became green fluorescent (Fig 3) after infection with JCPyV VLPs carrying the EGFP gene, indicating high-efficiency JCPyV VLP-mediated gene transfer into human lung adenocarcinoma cells. We also found that SPBtk-VLPs introduced into mice by tail vein injection were indeed able to reach subcutaneous human lung adenocarcinoma tumor nodules and cause them to shrink drastically in the presence of GCV (Fig 5). This finding shows that JCPyV VLPs can protect and deliver suicide gene to reach distant human lung adenocarcinoma cells through the blood circulation, thus achieving the desired therapeutic effect.

Lung cancer has not been a major target for gene therapy, since intratumoral injection of viral vectors is generally not feasible in the lungs. Further, lung adenocarcinomas tend to be located in the periphery of the lung, and has usually metastasized by the time it is diagnosed, thus necessitating systemic rather than local therapy. In contrast to the majority of gene therapy vectors for lung cancer developed thus far, which are applicable only locally (i.e., endobronchial) by intratumoral injection or inhalation administration, our current study showed that SPBtk-VLP may be administrated systemically for treating advanced or metastatic unresectable tumors in the lung [24, 37]. The high transduction efficiency of JCPyV VLPs, combined with the lung cancer–specific expression of an SP-B promoter–driven suicide gene, is likely to enhance the efficacy of lung cancer gene therapy and reduce off-target toxicity. The SP-B promoter has the additional advantage of being regulated by transcriptional factors and hormones; for instance, gene expression from the SP-B promoter is stimulated by dexamethasone [38] and inhibited by insulin [39]. Therefore, in the future, it is of interest to investigate the effect of adding dexamethasone to our gene therapy system to enhance the expression of the therapeutic transgene driven by the SP-B promoter.

## Conclusions

In this study, we have demonstrated that the JCPyV VLPs can be used as effective gene delivery vectors delivered by systemic circulation for gene therapy against human lung adenocarcinoma *in vitro* and *in vivo*. Selective cytotoxicity of a suicide gene driven by SP-B promoter enables targeted lung adenocarcinoma treatment. The JCPyV VLPs combined with the SP-B promoter may be used as transcriptionally targeted gene therapy vectors for treating human lung adenocarcinoma.
